# Effect of S-ketamine administered at the end of anesthesia on emergence delirium in preschool children undergoing tonsillectomy and/or adenoidectomy

**DOI:** 10.3389/fphar.2023.1044558

**Published:** 2023-02-17

**Authors:** Yang Chen, Feixiang Ru, Qiuping Ye, Xinzhe Wu, Xianwen Hu, Ye Zhang, Yun Wu

**Affiliations:** ^1^ Department of Anesthesiology and Perioperative Medicine, The Second Affiliated Hospital of Anhui Medical University, Hefei, China; ^2^ Department of Anesthesiology, Anhui Medical University, Hefei, China

**Keywords:** S-ketamine, emergence delirium, tonsillectomy, adenoidectomy, sevoflurane, preschool children

## Abstract

**Background:** S-ketamine (the S-isomer of ketamine) is twice as potent as the racemic mixture of this agent and carries fewer side effects when administered to humans. Information regarding the use of S-ketamine for the prevention of emergence delirium (ED) is limited. Thus, we evaluated the effect of S-ketamine administered at the end of anesthesia on ED in preschool children undergoing tonsillectomy and/or adenoidectomy.

**Methods:** We investigated 108 children aged 3–7 years, who were scheduled for elective tonsillectomy and/or adenoidectomy under general anesthesia. They were randomly assigned to receive either S-ketamine 0.2 mg/kg or an equal volume of normal saline at the end of anesthesia. The primary outcome was the highest score on the pediatric anesthesia ED (PAED) scale during the first 30 min post-surgery. The secondary outcomes included the incidence of ED (defined as a score of ≥ 3 on Aono scale), pain score, time to extubation, and incidences of adverse events. Multivariate analyses were also performed using logistic regression to evaluate the independent factors predictive of ED.

**Results:** The median (interquartile range) PAED score of the S-ketamine group (0 [0, 3]) was significantly lower than that in the control group (1 [0, 7]) (estimate median difference = 0, 95% confidence interval −2 to 0, *p* = 0.040). Significantly fewer patients in the S-ketamine group had an Aono scale score ≥ 3 (4 [7%] vs. 12 [22%], *p* = 0.030). Patients in the S-ketamine group also had a lower median pain score than did control subjects (4 [4, 6] vs. 6 [5, 8], *p* = 0.002). The time to extubation and incidences of adverse events were comparable between the two groups. However, multivariate analyses indicated that except S-ketamine use, pain scores, age and duration of anesthesia were independent factors predictive of ED.

**Conclusion:** S-ketamine (0.2 mg/kg) administered at the end of anesthesia effectively reduced the incidence and severity of ED in preschool children undergoing tonsillectomy and/or adenoidectomy without prolonging the time to extubation or increasing adverse events. However, S-ketamine use was not an independent factor predictive of ED.

## Introduction

Emergence delirium (ED) is a common complication that occurs in sevoflurane-anesthetized preschool children. It usually occurs during the early period of recovery from anesthesia, and its incidence in this population (up to 80%) is much higher than that among adults (Moore and Anghelescu, 2017). ED is mainly characterized by involuntary agitation with kicking, absence of eye contact with caregivers, inconsolability, and inability to recognize surroundings (Malarbi et al., 2011; Lin et al., 2016). These clinical manifestations not only affect the quality of recovery, but can also increase the risks of self-injury, surgical dehiscence, sleeping disorders, enuresis, and even persistent changes in emotional and cognitive functions (Kain et al., 2004; Malarbi et al., 2011; Mohkamkar et al., 2014). There have been many studies on the causes and/or prevention of ED; however, the pathogenesis of this condition remains unclear, and preventative treatments for it were ill-defined.

Various sedative and analgesic agents administrated systemically were found to be efficient in preventing ED. Ketamine is an N-methyl-D-aspartate receptor antagonist with a strong analgesic effect when administered at a dose lower than that required for anesthesia induction (Keidan et al., 2004). It has been reported that ketamine administered at such subanesthetic doses reduces the incidence of ED after rhinoplasty or dental repair (Abu-Shahwan and Chowdary, 2007; Demir and Yuzkat, 2018). However, the psychological adverse effects of ketamine (including nightmares and hallucinations) have hampered its deployment in clinical settings (Zanos et al., 2018).

S-ketamine, the left-handed optical isomer of the compound, is purported to have a higher potency and lower incidence of adverse events than racemic ketamine (Zhang et al., 2022). Therefore, we designed this randomized controlled trial to evaluate the effect of S-ketamine administered at the end of anesthesia on emergence delirium in preschool children undergoing tonsillectomy and/or adenoidectomy. The primary outcome was the pediatric anesthesia ED (PAED) scale (Wang et al., 2021a) during the first 30 min post-surgery. We hypothesized that S-ketamine would reduce the severity of ED in preschool children without increasing the incidences of post-anesthesia adverse events.

## Methods

### Study design and randomization

This prospective randomized controlled trial was conducted between January and July 2022; it was approved by the Ethics Committee of the Second Affiliated Hospital of Anhui Medical University (approval no.: YX2021-141) and was prospectively registered in the Chinese Clinical Trial Registry (http://www.chictr.org.cn, ChiCTR2200055477). The study was performed in accordance with the Consolidated Standards of Reporting Trials (CONSORT) criteria (Schulz et al., 2010) and complied with the Helsinki Declaration. Written informed consent was obtained from the parents of all participants.

Pediatric patients between 3 and 7 years of age with an American Society of Anesthesiologists physical status of I or II and who were scheduled for tonsillectomy and/or adenoidectomy requiring general anesthesia were enrolled. Children with a body mass index (BMI) of > 25 kg/m^2^, developmental delays, psychological or neurological disorders, abnormal airways, reactive airway disease, history of general anesthesia, history of previous allergies or known allergies to the current study’s drugs, history of chronic pain or recent administration of sedative and analgesic drugs were excluded from this study.

The enrolled patients were randomly assigned to either the control group or S-ketamine group using computer software at a 1:1 ratio. An assistant who was not involved in the study performed a blinded random allocation by preparing coded and sealed opaque envelopes. A nurse unaffiliated with patient care opened the envelopes shortly before the end of surgery and prepared study medication. The agent used for this study was diluted with 0.9% NaCl to yield a 2-mL syringe containing 5 mg/ml S-ketamine or 0.9% NaCl, which were identical in appearance and were labeled as “study medication” with patient number. Based on the assigned group, patients in the S-ketamine group received a bolus of intravenous S-ketamine (0.2 mg/kg) at the end of surgery. Patients in the control group received a bolus of 0.9% NaCl at the end of surgery. All patients, family members, outcome assessors, and clinical staff were blinded to the patients’ group allocation and did not have access to randomization until the data analysis was complete.

### Standard analgesia and anesthesia treatment

All patients fasted for 8 h with an opportunity to drink clear fluids up to 2 h before the operation. The subjects were allowed to stay with one of the caregivers in the holding area until entering the operating room. The preoperative anxiety at separation from the caregiver was assessed using a four-point behavior score: 1 = calm and cooperative, 2 = anxious but reassurable, 3 = anxious and not reassurable, and 4 = crying or resisting (Yuen et al., 2008; Cho et al., 2020). Subjects were taken to the operating theatre without premedication; upon arrival, they were monitored using non-invasive arterial pressure, pulse oximetry, capnography, and electrocardiography throughout the surgery. Anesthesia was induced *via* the inhalation of 8% sevoflurane with an oxygen inflow of 8 L/min using a face mask. Induction quality was briefly evaluated according to a four-point scale: 1 = crying and needing restraint; 2 = moderate fear that was assuaged with difficulty, 3 = slight fear but could be reassured easily, and 4 = asleep, calm, awake, and/or cooperative when accepting the mask (Köner et al., 2011; Kim et al., 2013). Once consciousness was lost, sevoflurane was adjusted to 3%–4% with an oxygen inflow of 2 L/min, and intravenous access was established. All patients received antiemetics with dexamethasone 0.15 mg/kg intravenously to prevent postoperative nausea and vomiting (PONV). Endotracheal intubation was then facilitated with intravenous sufentanil 0.2 μg/kg and cisatracurium 0.1 mg/kg–0.2 mg/kg.

After intubation, children were mechanically ventilated using the volume-controlled ventilation mode. The tidal volume was set to 6 mL/kg–8 mL/kg while the respiratory rate was set to 16 beats/min and further adjusted to maintain end-tidal carbon dioxide pressure between 35 mmHg and 45 mmHg. Anesthesia was maintained with inhalation of sevoflurane 2%–3%, which was discontinued approximately 5 min before the completion of surgery. Additionally, intravenous propofol (2 mg/kg/h–4 mg/kg/h) and remifentanil (0.2 μg/kg/min–0.3 μg/kg/min) were infused continuously until the end of surgery.

Upon the completion of the surgery, the oxygen flow was increased to 6 L/min to wash out residual sevoflurane in the alveoli. The study drug according to group allocation (either S-ketamine 0.2 mg/kg which was diluted in 0.9% NaCl or the 0.9% NaCl alone) was slowly administered intravenously using a 2 mL syringe. Extubation was performed after confirming regular breathing with sufficient tidal volume (> 5 mL/kg) and purposeful movement. After extubation, the patients were transferred to the post-anesthesia care unit (PACU).

### Postoperative assessment

Subjects were continuously monitored and cared for after their arrival in the PACU. Three investigators (one anesthesiologist and two nurses) who were blinded to the group allocation evaluated the ED and recovery as described previously (Kim et al., 2013). The incidence and degree of delirium was evaluated and recorded every 5 min during the first 30 min in the PACU, and the highest-recorded value was used for evaluation. The incidence of ED was evaluated by one of the nurses using the four-point agitation scale of Aono et al. (1 = calm; 2 = not calm but easily calmed; 3 = not easily calmed, moderately agitated, or restless; and 4 = combative, excited, or disoriented) (Ali and Abdellatif, 2013). ED was defined as an Aono scale score ≥ 3; subjects with scores ≥ 3 for more than 5 min were treated with intravenous propofol 1 mg/kg as a rescue medication. The anesthesiologist evaluated the severity of ED by using the PAED scale (Table 1) (Wang et al., 2021a). The final PAED score was derived by summing those of each items (range, 0–20); higher scores were indicative of a higher degree of ED. Additionally, a five-step ED scale (1 = obtunded with no response to stimulation, 2 = asleep but responsive to movement or stimulation, 3 = awake and responsive, 4 = crying, and 5 = thrashing behavior that requires restraint) (Bajwa et al., 2010) was also evaluated by another nurse.

**TABLE 1:** Pediatric Anesthesia Emergence Delirium scale.

Description of items	Scores				
	0	1	2	3	4
The child makes eye contact with the caregiver	Extremely	Very much	Quite a bit	Just a little	Not at all
The child’s actions are purposeful	Extremely	Very much	Quite a bit	Just a little	Not at all
The child is aware of the surroundings	Extremely	Very much	Quite a bit	Just a little	Not at all
The child is restless	Not at all	Just a little	Quite a bit	Very much	Extremely
The child is inconsolable	Not at all	Just a little	Quite a bit	Very much	Extremely

Moreover, the postoperative pain intensity was assessed by the anesthesiologist using the Children’s Hospital of Eastern Ontario pain scale (CHEOPS) (Table 2) every 5 min. The CHEOPS score is based on six items including crying, facial expression, verbal responses, torso movements, wound touching, and leg position; the score ranges from 4 to 13, with 4–6 indicating no pain while higher scores reflecting increasing pain intensity (Zhang et al., 2014; Quintão et al., 2021). Subjects with CHEOPS scores of 10 or higher were administrated intravenous dezocine 0.1 mg/kg.

**TABLE 2:** Children’s hospital of eastern Ontario pain scale.

Description of items	Scores			
	0	1	2	3
Crying		No cry	Moaning or crying	Scream
Facial expression	Smiling	Composed	Painful	
Verbal responses	No pain	No complaint of pain	Complaint of pain	
Torso movements		Relaxed	Nervous and quivering	
Wound touching		Not touching wound	Reach/Touch/Grab wound	
Leg position		Neutral	Squirm/Kicking/Tense	

All children were observed for at least 30 min in the PACU and were transferred to the ward when they became calm and met the modified Aldrete score of ≥ 9 (Aldrete, 2007). Patients were followed during the first 24 h post-surgery. The Aono scale score at 8 h and 24 h post-surgery was recorded, as were adverse events such as airway complications, oxygen desaturation (defined as an SpO_2_ < 90%), drowsiness, PONV, postoperative diplopia, hallucinations, or nightmares.

### Outcome measurements

The primary outcome was the highest PAED scale score recorded during the first 30 min post-surgery in the PACU. The secondary outcomes included the time to extubation (defined as the interval between discontinuing the anesthetic and extubation), incidence of Aono scale score ≥ 3, five-step ED scale score, CHEOPS score, length of PACU stay, and incidences of adverse events.

### Sample size and statistical analysis

The sample size was calculated based on the primary outcome (PAED score during the first 30 min in the PACU) using the PASS software version 15.0 for Windows (NCSS Statistical Software, LLC, Kaysville, Utah, United States). Based on the results of our pilot study wherein the primary outcome scores (means ± standard deviations [SD]) were 1.4 ± 1.8 for the S-ketamine group and 4.6 ± 5.5 for the control group, two simple *t*-tests were performed and the group allocation ratio was 1:1. Considering a power of 0.80 and an alpha error of 0.05, and assuming a loss to follow-up rate of 15%, the required sample size for each group was calculated as 54. Thus, a total of 108 patients were required for the study.

All statistical analyses were performed using SPSS (version 24.0; IBM Corp., Armonk, NY, United States). Kolmogorov-Smirnov tests and visual inspection of histograms were performed to test the normality of the data distribution. Continuous variables are expressed as means ± SDs or medians with interquartile ranges (IQRs), and inter-group differences were assessed for significance using independent *t*-tests for normally distributed data or the Mann-Whitney *U*-test for non-parametric data. Categorical variables are expressed as numbers (percentages), and inter-group differences were assessed using the chi-squared or Fisher’s exact tests in cases of expected frequencies < 5.

Multivariate analyses were performed using logistic regression with a forward selection algorithm. The following clinical factors that are potentially associated with ED (defined as an Aono scale score of ≥ 3) were subjected to the analyses: intervention (injection with S-ketamine or not), age, gender, BMI, PSAS score, induction cooperation degree, duration of anesthesia, time to extubation, and CHEOPS score. The nature of the hypothesis testing was 2-tailed, and a *p*-value < 0.05 was considered statistically significant.

## Results

**FIGURE 1:**
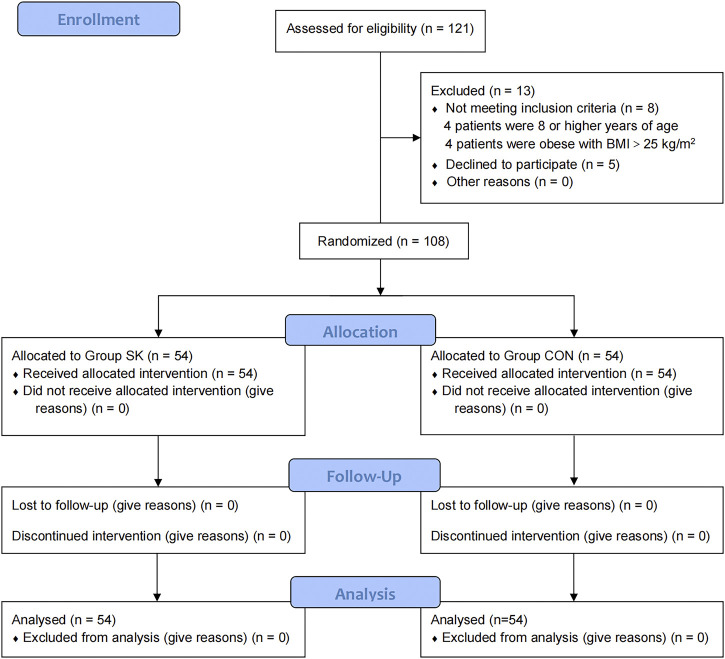
Consolidated Standards of Reporting Trials flow diagram of participants through each stage of the randomized trial.

The CONSORT flow diagram for this trial is shown in Figure 1. Patients were recruited between January 13 and 22 July 2022. While 121 children were initially screened for suitability, eight did not meet our inclusion criteria (four were ≥ 8 years of age and the remaining four were obese with BMI > 25 kg/m^2^); additionally, the parents of five children declined their participation in the study. Ultimately, 108 children were enrolled and randomized; all of whom received follow-up to the end of the study.

The patients’ characteristics and perioperative parameters are shown in Table 3. There were no significant differences in the overall characteristics, PSAS scores, induction quality, duration of anesthesia, and type of surgery between the two groups (Table 3).

**TABLE 3:** Patients’ characteristics and perioperative parameters.

Parameters	Group SK (*n* = 54)	Group CON (*n* = 54)	*p-*value
Age (yr)	5.0 (1.4)	5.4 (1.4)	0.181
Height (m)	1.2 (0.1)	1.2 (0.1)	0.264
Weight (kg)	22.3 (5.9)	22.3 (6.0)	0.994
BMI (kg/m^2^)	16.6 (3.0)	15.7 (2.6)	0.108
Gender			0.844
Male	33 (61.1)	32 (59.3)	
Female	21 (38.9)	22 (40.7)	
PSAS score	1.0 (1.0–1.0)	1.0 (1.0–1.0)	0.439
Induction quality	4.0 (3.0–4.0)	4.0 (3.0–4.0)	0.726
Duration of anesthesia (min)	48.7 (15.8)	43.5 (12.9)	0.067
Type of surgery			0.238
A	8 (14.8)	9 (16.7)	
B	10 (18.5)	17 (31.5)	
C	36 (66.7)	28 (51.8)	

Data are presented as means (standard deviations), medians (interquartile ranges) or numbers (percentages); Group SK, the S-ketamine group; Group CON, the control group; BMI, body mass index; PSAS, parental separation anxiety scale; A, tonsillectomy; B, adenoidectomy; C, tonsillectomy and adenoidectomy.

**FIGURE 2:**
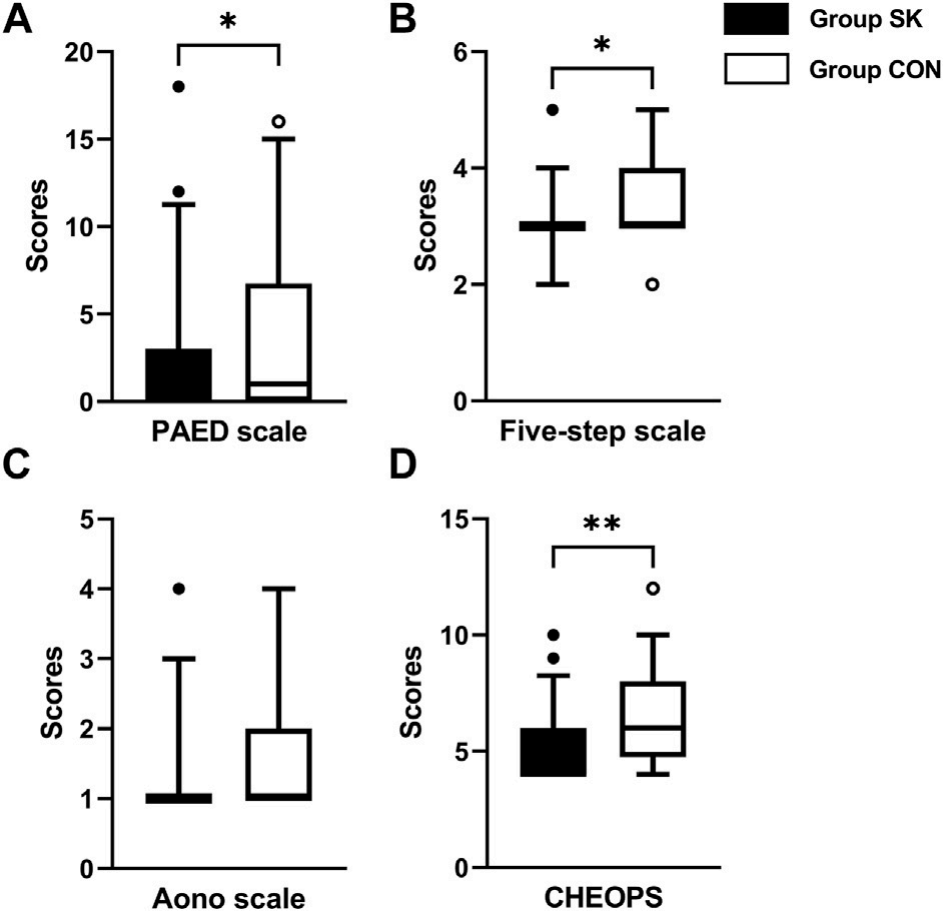
Postoperative assessment scores of PAED scale **(A)**, Aono scale **(B)**, five-step ED scale **(C)** and CHEOPS **(D)**. Data are expressed as median (horizontal bar), interquartile range (box), 5-95 percentile (whiskers) and outliers (circles). Inter-group differences were compared using Mann-Whitney *U* test. *p < 0.05 when compared with the control group. **p < 0.01 when compared with the control group. Group SK, the S-ketamine group; Group CON, the control group; PAED, pediatric anesthesia emergence delirium; CHEOPS, Children’s Hospital of Eastern Ontario pain scale.

During their PACU stay, the median (IQR) PAED score of the S-ketamine group (0 [0, 3]) was significantly lower than that of the control group (1 [0, 7]); the estimated median difference was 0 (95% confidence interval [CI]: -2 to 0, *p* = 0.040) (Figure 2A). Meanwhile, the five-step ED scale score was also significantly lower in the S-ketamine group than in the control group (median [IQR], 3 [3, 3] vs. 3 [3, 7]; estimate median difference = 0, 95% CI 0–0, *p* = 0.028) (Figure 2B). The median Aono scale scores of both groups were comparable (S-ketamine, 1 [1, 1] vs. control, 1 [1, 2]; estimate median difference = 0, 95% CI 0–0, *p* = 0.082) (Figure 2C). Patients in the S-ketamine group had a lower CHEOPS score than did those in the control group (4 [4, 6] vs. 6 [5, 8]; estimate median difference = 1, 95% CI 0–2, *p* = 0.002) (Figure 2D).

**FIGURE 3:**
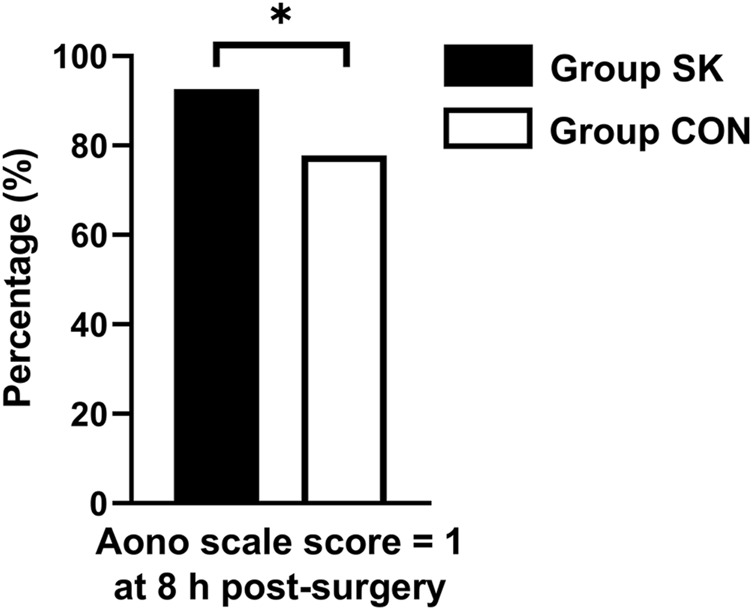
Distributions of patients with Aono scale = 1 at 8 h postoperatively. *p < 0.05 when compared with the control group. Group SK, the S-ketamine group; Group CON, the control group.

For the incidence of ED, the number (percentage) of patients with an Aono scale score ≥ 3 in the S-ketamine group (4 [7%]) was significantly lower than that in the control group (12 [22%]; *p* = 0.030). The time to extubation was comparable between the two groups (*p* = 0.236). Patients in the control group had significantly longer length of stay in the PACU than did those in the S-ketamine group (*p* = 0.008), while the nurses’ satisfaction scores for both groups were comparable (*p* = 0.070). Patients in both groups had similar incidences of PONV and drowsiness during the first 24 h post-surgery; however, none developed airway complications, oxygen desaturation, postoperative diplopia, hallucinations, or nightmares. The Aono scale score was significantly lower in the S-ketamine group at 8 h post-surgery than they were in the control group (*p* = 0.028), and all patients in both groups were calm at 24 h post-surgery (Table 4). Meanwhile, more patients of whom exhibited a calm status (Aono scale score = 1) at 8 h post-surgery than they were in the control group (*p* = 0.030) (Figure 3); Logistic regression analysis suggested that age, duration of anesthesia, and pain scores were independent predictors of ED; however, S-ketamine use was not shown to be an independent predictor of ED (Table 5).

**TABLE 4:** Postoperative conditions in two groups.

Parameters	Group SK (*n* = 54)	Group CON (*n* = 54)	*p-*value
Number of patients with Aono scale score ≥ 3	4 (7.41)*	12 (22.22)	0.030
Time to extubation (min)	12.4 (3.7)	13.7 (6.7)	0.236
Length of PACU stay (min)	32.0 (5.9)*	36.7 (11.1)	0.008
Nurses’ satisfaction score	9.3 (1.2)	8.8 (2.0)	0.070
Adverse events			
PONV	6 (11.1)	8 (14.8)	0.567
Drowsiness	2 (3.7)	3 (5.6)	> 0.999
Aono scale score at 8 h postoperatively	1.0 (1.0-1.0)*	1.0 (1.0–1.0)	0.028
Aono scale score at 24 h postoperatively	1.0 (1.0–1.0)	1.0 (1.0–1.0)	0.310

Data are presented as means (standard deviations), medians (interquartile ranges) or numbers (percentages), *p < 0.05, Significant difference compared with the control group; Group SK, the S-ketamine group; Group CON, the control group; PACU, post-anesthesia care unit; Length of PACU, stay, time interval between admission and discharge from PACU; PONV, postoperative nausea and vomiting.

**TABLE 5:** Binary logistic regression of clinical factors that are potentially associated with ED (defined as an Aono scale score of ≥ 3). Logistic regression analysis suggested that age, duration of anesthesia, and pain scores were independent predictors of ED.

Variables	B	SE	OR (95%CI)	*p-*value
Intervention				
Injection of normal saline				
Injection of S-ketamine	−1.731	1.167	0.177 (0.018–1.743)	0.138
Age	−1.058	0.487	0.347 (0.134–0.902)	0.030
Gender				
Female				
Male	−1.504	1.210	0.222 (0.021–2.381)	0.214
BMI	−0.077	0.199	0.926 (0.626–1.369)	0.699
PSAS score	−0.668	1.287	0.513 (0.041–6.389)	0.604
Induction cooperation degree	−0.938	0.864	0.392 (0.072–2.131)	0.278
Duration of anesthesia	0.113	0.046	1.120 (1.024–1.225)	0.013
Time to extubation	−0.124	0.135	0.884 (0.678–1.152)	0.360
CHEOPS score	1.390	0.408	4.014 (1.803–8.934)	0.001

Data of 108 participants; ED, emergence delirium; B, regression coefficient; SE, standard error; OR, odd radio; CI, confidence interval; PSAS, parental separation anxiety scale; CHEOPS, Children’s Hospital of Eastern Ontario Pain Scale.

## Discussion

Our data revealed that the administration of S-ketamine (0.2 mg/kg) at the end of anesthesia reduced the severity and incidence of ED in preschool children undergoing tonsillectomy and/or adenoidectomy without prolonging the time to extubation or increasing postoperative adverse events. Additionally, children who received S-ketamine experienced less pain and shorter PACU stay than those who did not.

Although the specific causes of ED following general anesthesia remain unclear, some of the reported risk factors include age, preoperative anxiety, type of surgery, duration of anesthesia, inhalation anesthetics, rapid awakening in an unfamiliar environment, and postoperative pain (Beskow and Westrin, 1999; Voepel-Lewis et al., 2005; Abu-Shahwan and Chowdary, 2007; Kanaya, 2016). Preschool children may be less able to cope with a strange environment upon rapidly awakening from sevoflurane anesthesia (Aono et al., 1997; Abu-Shahwan and Chowdary, 2007). Additionally, adenotonsillectomy is usually associated with a feeling of suffocation, postoperative pain, and bleeding; thus, preschool children undergoing tonsillectomy and/or adenoidectomy with sevoflurane anesthesia are more likely to experience ED (Przybylo et al., 2003), which increases the risk of postoperative airway obstruction and its associated complications. Therefore, a smooth emergence from general anesthesia as well as a calm recovery are critically important for anesthesiologists to ensure (Harris et al., 2016).

Ketamine is a highly lipid soluble N-methyl-D-aspartic ammonia acid receptor antagonist that has hypnotic effects at sub-anesthetic doses, causing the dissociation of the cortex from the limbic system. It does not cause respiratory depression at small doses (< 1 mg/kg), and has little effect on the heart rate and blood pressure (Jahangir et al., 1993). Various studies have demonstrated that ketamine at sub-anesthetic doses (0.25 mg/kg–0.5 mg/kg) is highly effective in preventing ED (Abu-Shahwan and Chowdary, 2007; Demir and Yuzkat, 2018; Fattahi-Saravi et al., 2021). The most commonly used ketamine in the clinic is a racemic mixture of two optical isomers, levo-ketamine (R-ketamine) and dextro-ketamine (S-ketamine). Owing to its reported psychotropic side effects, however, racemic ketamine use in the clinic has been reduced (White et al., 1982; Wang et al., 2021b). S-ketamine is twice as potent as the racemic mixture and is a preferable analgesic owing to its rapid onset of action and short half-life (Ihmsen et al., 2001); it reportedly achieves the same sedation efficiency as the racemic mixture at only 60% the dosage (Martin-Jurado et al., 2011), thereby causing fewer side effects (White et al., 1985; Nau and Strichartz, 2002). Therefore, S-ketamine can be an effective and safe alternative to racemic ketamine for perioperative use.

Given a previous study demonstrating that S-ketamine at 0.5 mg/kg–1.0 mg/kg could induce somnolence or sleep (van de Bunt et al., 2017), we evaluated emergence conditions 30 min after administering different doses of S-ketamine (between 0.1 mg/kg and 1.0 mg/kg) in our own pilot study and observed that smaller-dose regimens were more suitable. Pharmacological agents administered upon the completion of anesthesia are not dependent on the duration of surgery or anesthesia, or on the characteristics of anesthetic drugs previously administered during induction and maintenance (Abu-Shahwan and Chowdary, 2007; Aouad et al., 2007; Dahmani et al., 2010). Meanwhile, Han et al. (2022b) demonstrated that S-ketamine can be safely and effectively used as an adjuvant in patient-controlled intravenous analgesia without increasing complications such as PONV, dizziness, and respiratory depression. Thus, we chose to administer 0.2 mg/kg of S-ketamine at the end of anesthesia. As hypothesized, the PAED score in the S-ketamine group was significantly lower than that in the control group; additionally, significantly fewer patients had an Aono scale score of ≥ 3. These data indicated that administering S-ketamine at the end of anesthesia significantly reduced the incidence and severity of ED in preschool children undergoing tonsillectomy and/or adenoidectomy. Since a greater number of patients with an Aono scale score of 3 or higher received propofol as a rescue treatment, we speculated that a higher incidence of ED led to longer monitoring and PACU stay.

Increased pain has been identified as a possible cause of ED associated with pediatric surgery (Galinkin et al., 2000). Owing to their potent analgesic and sedative properties, opioids were demonstrated to successfully reduce the incidence of ED by pain-relief in children; however, this was accompanied by a higher incidence of PONV, delayed removal of the airway device, and longer stay in the PACU. Moreover, airway-related adverse events such as suspicious laryngospasm and respiratory depression were also observed (Shi et al., 2015; Kim et al., 2017). Conversely, adverse respiratory events are rare when using S-ketamine owing to protective airway reflexes, and studies have also demonstrated that perioperative intravenous S-ketamine significantly reduces postoperative pain without increasing the risk of PONV (Wang et al., 2021b). Similarly, our results showed that the CHEOPS score in the S-ketamine group was significantly lower than that in the control group, with no respiratory adverse events occurring. Furthermore, the incidences of PONV were comparable between the two groups, which is consistent with the analgesic characteristics of S-ketamine. We also found that patients with lower CHEOPS scores exhibited lower PAED scores, and multivariate analyses with logistic regression indicated that the pain scores, along with age and duration of anesthesia, were independent factors predictive of ED, thereby suggesting that pain relief was associated with a lower incidence of ED. However, we did not find that S-ketamine use was an independent factor in ED prevention; our sample size was calculated based on decreasing the PAED, and may not have been sufficiently large to address ED prevention *per se*.

A reliable scale or scoring system for assessing the incidence and severity of ED should be used for an objective comparison between two groups. The PAED scale is one such reliable and validated tool for evaluating ED in children (Han et al., 2022a); however, ED assessment might be inconsistent when using different evaluation tools (Bajwa et al., 2010; Dahmani et al., 2010). Therefore, we also used the five-step ED scale in addition to the PAED scale, whereby S-ketamine was still found to be effective. Meanwhile, Aono scale is another reliable scale system for assessing the incidence of ED and an Aono scale score ≥ 3 had been defined as the ED (Kim et al., 2013). In our study, there were less patients exhibited an Aono scale score ≥ 3 in the S-ketamine group. Additionally, some patients experienced ED in the later postoperative period, and S-ketamine also appeared to help prevent such incidences given that a greater proportion of patients in the S-ketamine group had Aono scale scores of 1 at 8 h post-surgery. Different evaluation tools further confirm the reliability of the results. Thus, we can expect that even when using a small dosage bolus, S-ketamine promises to be effective during recovery after pediatric surgery.

Our study had several limitations. First, we investigated only children with otorhinolaryngological surgeries, and we excluded the children with BMI > 25 kg/m^2^; given that the incidence of ED can differ depending on the type of surgery and BMI, our inclusion and exclusion criteria limited the generalizability of the results. Second, we did not evaluate behavioral changes for longer than 24 h post-surgery. Third, the sedative effect of sufentanil and the hyperalgesic effect of remifentanil would both affect the incidence and severity of ED. Forth, this was a single-center study, and whether single-dose S-ketamine can prevent ED in other settings remains to be ascertained.

In summary, our data demonstrated that S-ketamine at 0.2 mg/kg administered at the end of anesthesia safely and effectively reduces the incidence and severity of ED as well as postoperative pain in preschool children undergoing tonsillectomy and/or adenoidectomy. However, S-ketamine use was not an independent factor predictive of ED.

## Data Availability

The raw data supporting the conclusion of this article will be made available by the authors, without undue reservation.
